# Measurable Residual Disease Monitoring by Locked Nucleic Acid Quantitative Real-Time PCR Assay for *IDH1/2* Mutation in Adult AML

**DOI:** 10.3390/cancers14246205

**Published:** 2022-12-15

**Authors:** Hsiao-Wen Kao, Ming-Chung Kuo, Ying-Jung Huang, Hung Chang, Shu-Fen Hu, Chein-Fuang Huang, Yu-Shin Hung, Tung-Liang Lin, Che-Wei Ou, Ming-Yu Lien, Jin-Hou Wu, Chih-Cheng Chen, Lee-Yung Shih

**Affiliations:** 1 Division of Hematology-Oncology, Department of Internal Medicine, Chang Gung Memorial Hospital at Linkou, Taoyuan 333423, Taiwan; 2 College of Medicine, Chang Gung University, Taoyuan 333323, Taiwan; 3 Division of Hematology and Oncology, Department of Internal Medicine, China Medical University Hospital, Taichung 404327, Taiwan; 4 Division of Hematology and Oncology, Department of Internal Medicine, Chang Gung Memorial Hospital at Chiayi, Chiayi 613016, Taiwan

**Keywords:** acute myeloid leukemia, *IDH1/2* mutation, measurable residual disease, *NPM1* mutation, locked nucleic acid quantitative PCR

## Abstract

**Simple Summary:**

Measurable residual disease (MRD) monitoring is crucial in managing AML to predict the risk of relapse. A better understanding of which MRD technique and molecular target will have an effective clinical impact on AML is still required. Locked nucleic acid quantitative Real-Time PCR assay (LNA-qPCR) is sensitive and specific for quantifying oncogenetic single-nucleotide variation. We assessed the role of LNA-qPCR in the monitoring of *IDH1/2* mutations MRD in eighty-eight AML patients from multiple centers. We found that *IDH1/2* LNA-qPCR MRD correlates well with *NPM1* qPCR MRD, predicts relapse-free survival and cumulative incidence of relapse, and is a potential MRD technique for *IDH1/2-*mutated AML patients with reduced *IDH1/2* mutant levels after complete remission.

**Abstract:**

Locked nucleic acid quantitative Real-Time PCR (LNA-qPCR) for *IDH1/2* mutations in AML measurable residual disease (MRD) detection is rarely reported. LNA-qPCR was applied to quantify *IDH1/2* mutants MRD kinetics in bone marrow from 88 *IDH1/2-*mutated AML patients, and correlated with *NPM1*-MRD, clinical characteristics, and outcomes. The median normalized copy number (NCN) of *IDH1/2* mutants decreased significantly from 53,228 (range 87–980,686)/*ALB* × 10^6^ at diagnosis to 773 (range 1.5–103,600)/*ALB* × 10^6^ at first complete remission (CR). *IDH1/2* LNA-qPCR MRD was concordant with remission status or *NPM1*-MRD in 79.5% (70/88) of patients. Younger patients and patients with *FLT3* mutations had higher concordance. The Spearman correlation coefficient (r_s_) and concordance rate between the log reduction of *IDH1/2* LNA-qPCR and *NPM1*-MRD were 0.68 and 81% (*K* = 0.63, 95% CI 0.50–0.74), respectively. *IDH1/2*-MRD > 2 log reduction at first CR predicted significantly better relapse-free survival (3-year RFS rates 52.9% vs. 31.9%, *p* = 0.007) and cumulative incidence of relapse (3-year CIR rates 44.5% vs. 64.5%, *p* = 0.012) compared to *IDH1/2-*MRD ≤ 2 log reduction. *IDH1/2*-MRD > 2 log reduction during consolidation is also associated with a significantly lower CIR rate than *IDH1/2*-MRD ≤ 2 log reduction (3-year CIR rates 42.3% vs. 68.8%, *p* = 0.019). LNA-qPCR for *IDH1/2* mutation is a potential MRD technique to predict relapse in *IDH1/2-*mutated AML patients, especially for those with *IDH1/2* MRD > 2 log reduction at first CR or a concurrent *FLT3* mutation.

## 1. Introduction

Acute myeloid leukemia (AML) is a hematological malignancy characterized by clonal expansion of myeloid progenitors in the bone marrow (BM), peripheral blood, and other tissues. AML is a heterogeneous disease in terms of molecular genetics and phenotypic characteristics [1,2]. Molecular screening plays a significant role in prognostic categorization and decision of treatment strategies for AML [3].

Isocitrate dehydrogenases 1 and 2 (IDH1, IDH2) are metabolic enzymes that convert isocitrate to α-ketoglutarate, which are involved in diverse cellular processes, including adaptation to hypoxia, histone demethylation, and DNA modification [4,5]. Somatic mutations in *IDH1* and *IDH2* genes cause their protein dysfunction and an intracellular accumulation of aberrant 2-hydroxy-glutarate, a cancer-associated metabolite [6,7]. The incidence of *IDH1/2* mutations in AML was 6%–14% for *IDH1* [8,9,10,11], and 8%–19% for *IDH2* [8,10,12]. Most of these mutations involve hot-spot codons R140 and R172 in exon 4 of *IDH2* and codon R132 in exon 4 of *IDH1*. The prognostic impact of *IDH1/2* mutations is still controversial. Mutated *IDH1*-R132 has been reported as an unfavorable [9,11] or non-significant factor for AML outcome [8,10]. Mutated *IDH2*-R140 has been described as favorable or with no impact on overall survival [10,13], whereas mutated *IDH2*-R172 was associated with a worse prognosis [9,13].

Minimal or measurable residual disease (MRD) has prognostic value in AML patients during or after treatment [14,15,16,17]. The use of *IDH1/2* mutations as molecular markers for MRD monitoring is still under investigation. A few studies have reported the stability and suitability of *IDH1/2* mutations to monitor MRD by digital PCR (ddPCR) [18,19], next-generation sequencing (NGS) [20,21,22], and locked nucleic acid (LNA) quantitative Real-Time PCR (LNA-qPCR) [23,24]. Persistent *IDH1* or *IDH2* mutations have been observed in AML patients at the time of morphological remission [22,25]. Two studies with large cohorts of patients have reported that persistent mutated *IDH1/2* MRD detected by ddPCR or NGS in AML at the time of complete remission (CR) could predict relapse [19,20]. The impact of *IDH1/2* MRD by LNA-qPCR on the outcome of AML patients has not been investigated in large cohorts previously.

*NPM1* mutation is a stable MRD marker and *NPM1* PCR assays are recommended in AML patients with *NPM1* mutation [14,17,26]. In the present study, we aimed to evaluate the use of LNA-qPCR assay to monitor MRD after treatment in AML patients harboring *IDH1*-R132, *IDH2*-R140, or *IDH2*-R172 mutations, to correlate the MRD results of *IDH1/2* mutations with those of *NPM1* mutations, and to determine their impact on the outcome.

## 2. Materials and Methods

### 2.1. Patients and Samples

Newly diagnosed adult de novo AML patients who received induction therapy, including standard chemotherapy, azacitidine, or low-dose cytarabine, were enrolled. This study was conducted by the AML consortium of Taiwan, which aimed to detect genetic mutations and monitor MRD for AML patients in Taiwan. BM samples at the initial diagnosis were analyzed for cytogenetics and a panel of gene mutations. G-banding method was used for karyotypic analysis. AML classification and risk categorization followed the criteria of 2022 WHO classification and 2022 ELN recommendations [3,27]. The study was approved by the Institutional Review Board of Chang Gung Memorial Hospital (99-0112B, 102-3031B, 201700154B0, and 201701453A3) and the participating hospitals (CMUH-HO-AML-10701). Informed consent was obtained from enrolled patients.

BM samples were collected at diagnosis and at different time points after treatment, including the first CR documented after induction therapy, during consolidation (after one to two cycles of consolidation therapy), end of treatment, and relapse. Mononuclear cells were enriched by using Ficoll density gradient centrifugation and cryopreserved. Genomic DNA (gDNA) or RNA extraction and cDNA preparation were performed as previously described [28].

### 2.2. Detection of Gene Mutations

Diagnostic BM samples were examined for the presence of *IDH1* and *IDH2* mutations by using NGS (n = 64, MiSeq with SOPHiA DDM™ for Blood Cancers), pyro-sequencing (n = 35), and/or direct sequencing (n = 4). Mutations of *NPM1*, *CEBPA*, *FLT3-*ITD, *FLT3-*TKD, and myelodysplasia-related gene mutations were detected by NGS or methods described previously [28].

### 2.3. Monitoring of IDH1/2 MRD by LNA-qPCR Assay

**Figure 1:**
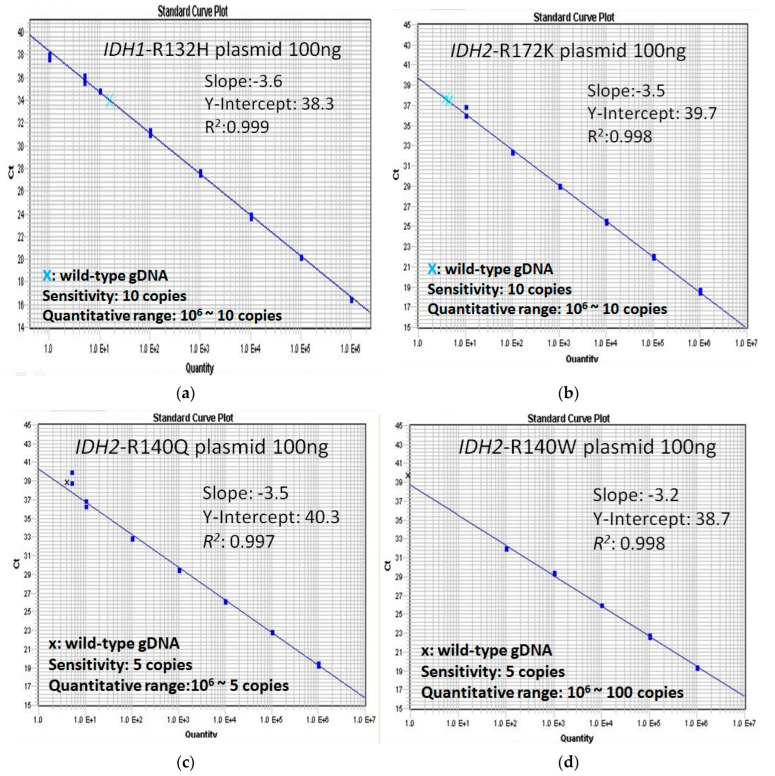
Standard curves constructed by *IDH1/2* mutant plasmids. (**a**) *IDH1*-R132H. (**b**) *IDH2*-R172K. (**c**) *IDH2*-R140Q. (**d**) *IDH2*-R140W.

Quantification of *IDH1/2* mutation copy number was assessed by quantitative Real-Time PCR (qPCR) for gDNA using the TaqMan technology with LNA probes specific for the mutant allele to increase the specificity for single-nucleotide variation in *IDH1*-R132, *IDH2*-R140, and *IDH2*-R172. The primer sequences and DNA probes used to amplify *IDH1-*R132, *IDH2-*R140, and *IDH2-*R172 mutations are shown in Table 1. Probes, forward primers, and reverse primers for *IDH2*-R140Q, *IDH2*-R140W, *IDH1-*R132H, *IDH1-*R132S, *IDH1-*R132G, and *ALB* were the same as those previously reported [24,29]. The reverse primer of *IDH2-*R172K was modified by removing the R-LNA. Reverse primers of *IDH1-*R132C and *IDH1-*R132L were designed in our lab. A locked nucleic acid was fixed in the last 3′nucleotide of the reverse primers. Reaction mixtures of 25 µL contained the 100 ng gDNA, 2 × TaqMan Master Mix buffer (ThermoFisher), 0.08% BSA, 400 nM of each primer, and 200 nM probe. The PCR cycling protocol consisted of 2 min at 50 °C, 10 min of initial denaturation at 92 °C, and was followed by 50 cycles of 15 s at 95 °C, 1 min at 58 °C. *Albumin* (*ALB*) was used as a reference gene. *IDH1/2* standard curves were constructed by serial dilution of *IDH1*-R132, *IDH2-*R140, and *IDH2-*R172 plasmids with wild-type *IDH1/2* gDNA from normal peripheral blood (Figure 1a–d). Specificity was determined by negative control with wild-type *IDH1/2* gDNA. The sensitivity of the assay was five copies for *IDH1*-R132C/S/G/L, ten copies for *IDH1*-R132H, five copies for *IDH2*-R140Q/W, and ten copies for *IDH2*-R172K with a quantitative range from 5–100 to 10^6^ copies. The normalized copy number (NCN) of the *IDH1/2* mutant of the sample was determined by the *IDH1/2-*mutant copies/*ALB* × 10^6^ copies. The result of *IDH1/2* MRD was expressed as log reduction by calculation of NCN^follow-up^ divided by NCN^diagnosis^ for the individual patients. The mean NCN (*IDH1/2* copies/*ALB* × 10^6^ copies) of 19 BM samples from healthy donors were 1119.4 for R132C, 0.2 for R132G, 462.2 for R132H, 11.3 for R132L, 77.2 for R132S, 337.3 for R140Q, 122.5 for R140W, and 81.1 for R172K (Table S1, Figure S1). The MRD negativity is defined as a NCN level that is less than the mean NCN of normal BM samples for each *IDH1/2* mutation type.

### 2.4. Detection of NPM1 RT-qPCR MRD

*NPM1* MRD was detected by quantitative Real-Time reverse transcriptase PCR (RT-qPCR) with the TaqMan assay using *ABL1* as the internal control gene. Primers and probes used were according to the method described by Gorello et al. [30]. The MRD results were expressed as a log reduction of the NCN at follow-up compared to the NCN at diagnosis.

### 2.5. Statistical Analysis

Wilcoxon’s rank-sum test, Fisher’s exact test, and the χ^2^ analysis were used whenever appropriate to make comparisons between groups. The correlation between *IDH1/2* LNA-qPCR, marrow blasts, and *NPM1* RT-qPCR was assessed by Spearman’s rank correlation test. Cohen’s kappa (*K*) was used to evaluate the concordance between *IDH1/2* LNA-qPCR and *NPM1* RT-qPCR. The *IDH1/2* MRD at first CR after induction therapy and during consolidation was subjected for outcome analysis. The overall survival (OS), relapse-free survival (RFS), and cumulative incidence of relapse (CIR) are based on the 2022 ELN AML recommendation [3]. Estimates of survival were calculated according to the Kaplan–Meier method. The log-rank test analyzed the differences in survival for significance. Differences in the CIR were assessed using the Gray test. In all analyses, the *p*-values were two-sided and considered statistically significant when values < 0.05. Statistical analysis was carried out by R version 4.1.3, R Foundation for Statistical Computing, Vienna, Austria.

## 3. Results

### 3.1. Patient Characteristics

Eighty-eight *IDH1/2-*mutated AML patients with post-treatment follow-up BM samples were enrolled for MRD evaluation. There were 25 *IDH1* mutations (11 R132C, 9 R132H, 2 R132G, 2 R132S, 1 R132L) and 63 *IDH2* mutations (49 R140Q, 2 R140W, 12 R172K). The median age was 54 (range 18–86) years. Cytogenetic normal, intermediate, and adverse risk groups based on the 2022 ELN AML recommendation account for 64%, 17%, and 9%, respectively (10% no metaphase). Eighty-four (95%) patients received standard “3 + 7” chemotherapy, two (2%) patients received azacitidine, and two (2%) patients received low-dose cytarabine as induction therapy. Thirteen patients received allogeneic hematopoietic stem cell transplantation (HSCT). Forty-two (48%) patients had concurrent *NPM1* mutations. The detailed clinical and genetic characteristics of patients are summarized in Table 2.

### 3.2. NCN of IDH1/2 Mutations in Pre-Treatment and Post-Treatment BM Samples

**Figure 2:**
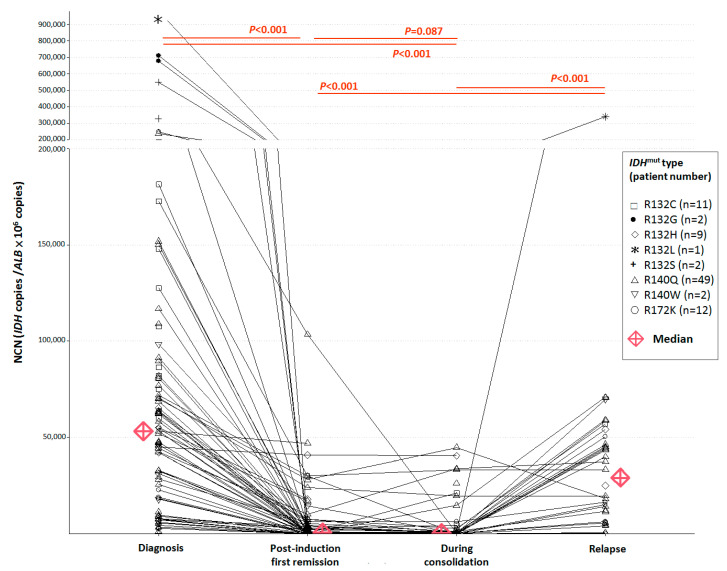
Changes in normalized copy numbers of *IDH1/2* mutants at diagnosis, post-induction first complete remission, during consolidation therapy, and relapse.

The median diagnostic NCN was 80,920 (31,343–980,686)/*ALB* × 10^6^ for 25 *IDH1-*mutated patients and 46,156 (87–247,796)/*ALB* × 10^6^ for 63 *IDH2-*mutated patients. *IDH1/2-*NCN at diagnosis (median 53,228, range 87–980,686/*ALB* × 10^6^) was significantly higher than both *IDH1/2*-NCN at first CR (median *IDH1/2* NCN at first CR 773, range 1.5–103,600/*ALB* × 10^6^*, p <*< 0.001) and *IDH1/2*-NCN during consolidation therapy (median 299, range 0–44,828/*ALB* × 10^6^, *p <*< 0.001). There was no significant difference between *IDH1/2*-NCN at first CR and *IDH1/2*-NCN during consolidation therapy (*p* = 0.087). *IDH1/2-*NCN at relapse from 34 patients (median 29,253, range 364–339,239/*ALB* × 10^6^, *p <*< 0.001) was significantly higher than *IDH1/2*-NCN at first CR and during consolidation therapy (Figure 2).

### 3.3. Correlation of IDH1/2 LNA-qPCR MRD with NPM1 RT-qPCR MRD and Hematologic Remission Status

Of the 25 *IDH1-*mutated AML patients, nine had *NPM1* mutation RT-qPCR MRD for comparison. Of the nine coexisting *NPM1* and *IDH1* mutated patients, six had good correlations among *IDH1* LNA-qPCR MRD, *NPM1* RT-qPCR, and marrow blast percentage (Figure S2), while three had persistently high *IDH1* LNA-qPCR MRD (≤2 log reduction) but with low *NPM1* RT-qPCR MRD and marrow blasts <5% (Figure S3a). Of the 16 *IDH1-*mutated patients without *NPM1* mutation, eight patients with marrow blasts <5% had *IDH1* LNA-qPCR MRD > 2 log reduction (Figure S4), four patients with refractory AML and marrow blasts >5% (ranged 9.7% to 59.3%) had *IDH1* LNA-qPCR MRD ≤ 2 log reduction (Figure S5), and four patients with marrow blasts <5% had persistent *IDH1* qPCR MRD ≤ 2 log reduction (Figure S3b). The concordance rate of *IDH1* LNA-qPCR MRD with remission status and/or *NPM1* MRD was 72.0% (18/25).

Among the 63 *IDH2-*mutated AML patients, 22 had concurrent *NPM1* mutation RT-qPCR MRD for comparison, 17 had good concordance between *IDH2* LNA-qPCR MRD, *NPM1* RT-qPCR (Figure S6), and marrow blasts, while five patients had persistently high *IDH2* LNA-qPCR MRD but with low *NPM1* RT-qPCR MRD and marrow blasts <5% (Figure S7a). Of the 41 *IDH2-*mutated patients without *NPM1* mutation, 29 patients had good concordance between serial follow-up for *IDH2* LNA-qPCR MRD and the percentage of marrow blasts (Figure S8). Of note, *IDH2* LNA-qPCR MRD elevations preceded frank hematologic relapse (Figure S8. #24, #50, #59). Six patients with marrow blasts >5% had *IDH2* qPCR MRD ≤ 2 log reduction (Figure S9), but another six patients with marrow blasts <5% had persistent *IDH2* qPCR MRD ≤ 2 log reductions (Figure S7b). The concordance rate of *IDH2* LNA-qPCR MRD with remission status and/or *NPM1* MRD was 82.5% (52/63).

Of the 25 relapsed patients with good concordance between *IDH1/2* LNA-qPCR MRD and diagnosis-remission status, all patients retained *IDH1/2* mutation at relapse, and the stability of *IDH1/2* LNA-qPCR at AML relapse was 100% (25/25). The *IDH1/2* LNA-qPCR MRD kinetics are summarized in Figure S10 based on concordance and remission status. Overall, the concordance rate of *IDH1/2* LNA-qPCR MRD with remission status or *NPM1* MRD was 79.5% (70/88).

### 3.4. Correlation between IDH1/2 LNA-qPCR MRD, NPM1 RT-qPCR, and Marrow Blasts in Serial BM Samples

**Figure 3:**
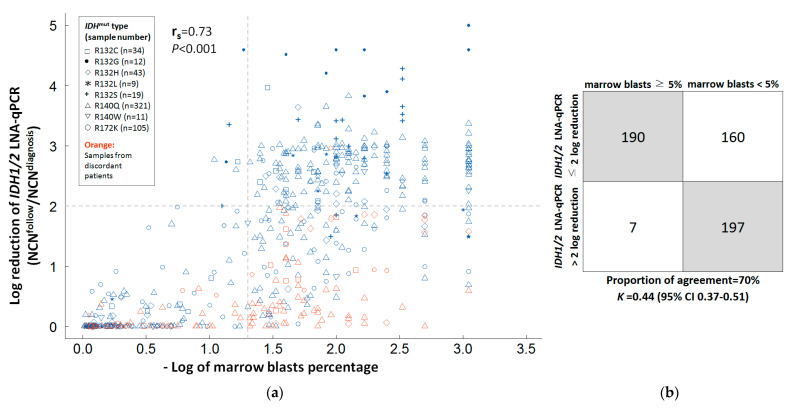
Correlation and agreement between *IDH1/2* LNA-qPCR MRD and marrow blasts in serial bone marrow samples. (**a**) Correlation between *IDH1/2* LNA-qPCR MRD and marrow blasts by log reduction. Gray dashed lines showed two-log reduction of *IDH1/2* LNA-qPCR and 1.3-log (5%) of marrow blasts percentage. Orange points represent samples from patients with discordant reduction of *IDH1/2* LNA-qPCR in complete remission status. Blue points represent samples from patients with regeneration marrow. (**b**) Agreement between *IDH1/2* LNA-qPCR MRD and marrow blasts of all samples.

Of 554 serial BM samples from all patients (median sample number per patient 5.5, range 2–16), there was a positive correlation between the log reduction of *IDH1/2* LNA-qPCR and BM blasts with Spearman correlation coefficient (r_s_) of 0.73 (*p* << 0.001, Figure 3a). The proportion of concordance was 70% (*K* = 0.44, 95% CI 0.37–0.51) between log reduction of *IDH1/2* LNA-qPCR (≤2 log or >2 log) and BM blasts (≥5% or <5%) in all samples (Figure 3b).

**Figure 4:**
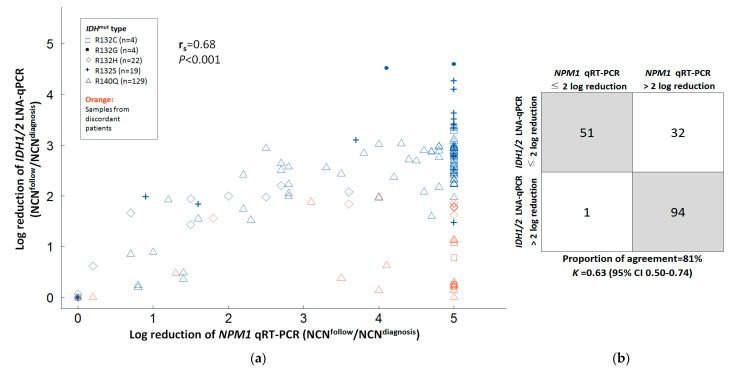
Correlation and agreement between *IDH1/2* LNA-qPCR MRD and *NPM1* RT-qPCR MRD in serial bone marrow samples. (**a**) Correlation between *IDH1/2* LNA-qPCR and *NPM1* RT-qPCR MRD by log reduction. Gray dashed lines showed two-log reduction of *IDH1/2* LNA-qPCR and *NPM1* RT-qPCR MRD. Orange points represent samples from patients with discordant reduction of *IDH1/2* LNA-qPCR in complete remission status. (**b**) Agreement between *IDH1/2* LNA-qPCR and *NPM1* RT-qPCR MRD for all samples.

In 178 serial BM samples from 25 patients with concurrent *NPM1* mutation (median sample number per patient 4.5, range 2–12), there was a positive correlation between the log reduction of *IDH1/2* LNA-qPCR and *NPM1* RT-qPCR MRD with r_s_ of 0.68 (*p* << 0.001, Figure 4a). The concordance rate was 81% (*K* = 0.63, 95% CI 0.50–0.74) between log reduction of *IDH1/2* LNA-qPCR (≤2 log or >2 log) and *NPM1* RT-qPCR (≤2 log or >2 log) MRD in all samples (Figure 4b).

### 3.5. Correlation of IDH1/2 LNA-qPCR MRD with Clinical and Genetic Characteristics of AML Patients

The difference in MRD results between *NPM1* RT-qPCR MRD and *IDH1/2* LNA-qPCR methods was analyzed for patients with good correlations and *IDH1/2* LNA-qPCR MRD > 2 log reduction. The median log reduction of MRD was 5 log (range 2.7–5.0) by *NPM1* RT-qPCR and 2.9 (range 2.0–4.6) by *IDH1/2* LNA-qPCR. The level of MRD log reduction was significantly higher by *NPM1* RT-qPCR than by *IDH1/2* LNA-qPCR (*p* << 0.001), with a median difference of 2.0 log (range −0.4–2.8).

Patients with a good correlation between *IDH1/2* LNA-qPCR MRD and AML remission status were significantly younger than patients without correlation (median age 53 vs. 61 years, *p* = 0.025). Patients with *FLT3* mutations (either *FLT3*-ITD or *FLT3*-TKD) had significantly higher concordance rates between *IDH1/2* LNA-qPCR MRD and AML remission status than patients without *FLT3* mutations (96% vs. 71%, *p* = 0.012). The diagnostic *IDH1/2* NCN, white blood cell counts, hemoglobin, platelet counts, percentages of circulating or marrow blasts, and the mutation status of *NPM1* had no effect on the correlation between *IDH1/2* LNA-qPCR MRD and AML remission status (Table 3).

**Figure 5:**
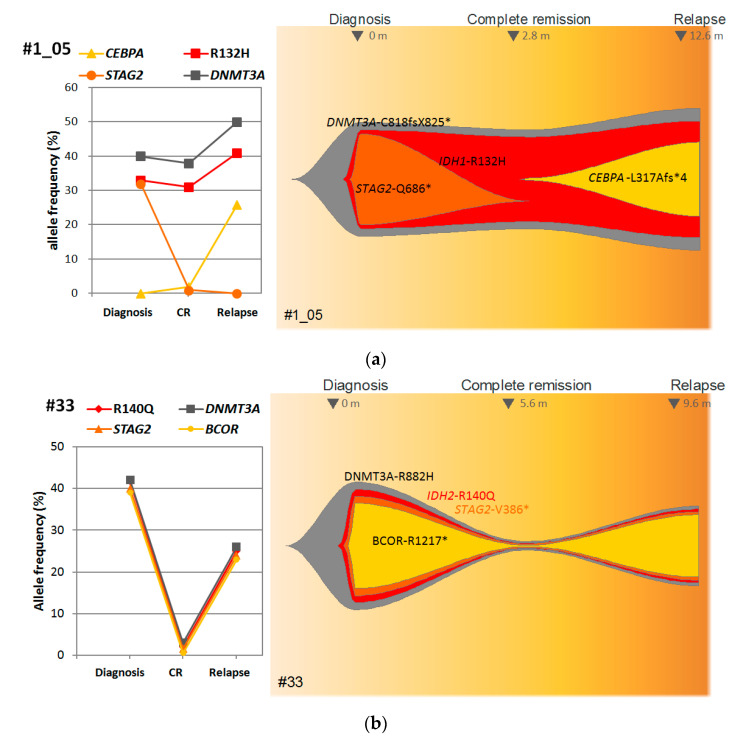
Changes in allele frequency of concurrent mutations (**left**) and hypothesized clonal evolution (**right**) at diagnosis, complete remission, and relapse of AML. (**a**) A patient with persistently high *IDH1*-R132H MRD, and (**b**) a patient with concordant *IDH2*-R140Q MRD.

To explore the clonal evolution of *IDH1/2* mutated AML patients in the different stages, we detected co-mutations with allele frequencies in one patient with discordant *IDH1*-R132H MRD and another patient with concordant *IDH2*-R140Q MRD at the diagnosis, CR, and relapse samples. The patient with discordant *IDH1*-R132H MRD had stable clone sizes of *DNMT3A* mutation and *IDH2*-R140Q at diagnosis, CR, and relapse, but lost *STAG2* mutation and acquired *CEBPA* mutation at relapse (Figure 5a). Another patient with concordant *IDH2*-R140Q had reduced allele frequency of *IDH2*-R140Q and other co-mutations at CR, but *IDH2*-R140Q and co-mutations at diagnosis re-expanded at relapse (Figure 5b).

### 3.6. Outcome Impact of IDH1/2 LNA-qPCR MRD at the End of Induction

**Figure 6:**
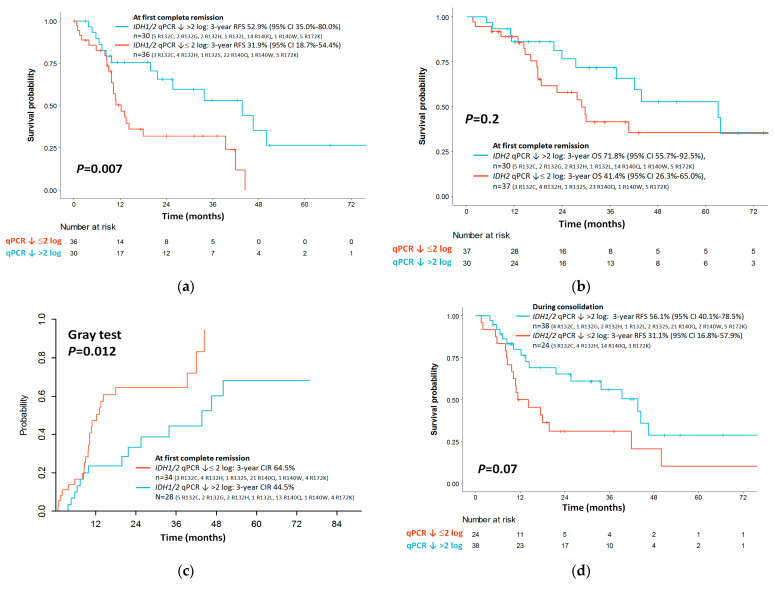

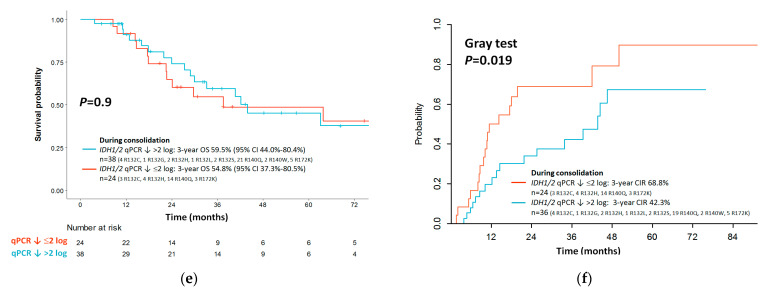
The relapse-free survival (**a**), overall survival (**b**), and cumulative incidence of relapse (**c**) of AML patients based on *IDH1/2* LNA-qPCR MRD at post-induction of first complete remission. The relapse-free survival (**d**), overall survival (**e**), and cumulative incidence of relapse (**f**) of AML patients based on *IDH1/2* LNA-qPCR MRD during consolidation therapy.

For patients at first CR after induction therapy, those who achieved *IDH1/2* LNA-qPCR MRD > 2 log reduction had significantly better RFS compared to patients with *IDH1/2* LNA-qPCR MRD ≤ 2 log reduction (3-year RFS rates 52.9% [95% CI 35.0%–80.0%] vs. 31.9% [95% CI 18.7%–54.4%], *p* = 0.007, Figure 6a). *IDH1/2* LNA-qPCR MRD > 2 log reduction did not predict a longer OS than patients with *IDH1/2* LNA-PCR MRD ≤ 2 log reduction (3-year OS rates 71.8% [95% CI 55.7%–92.5%] vs. 41.4% [95% CI 26.3%–65.0%], *p* = 0.2; Figure 6b). A significantly higher CIR was observed for patients with *IDH1/2* LNA-qPCR MRD ≤ 2 log reduction than patients with *IDH1/2* qPCR MRD > 2 log reduction (3-year CIR 64.5% vs. 44.5%, *p* = 0.012; Figure 6c).

Based on *IDH1/2* mutation subtypes, *IDH2*-R140 post-induction MRD ≤ 2 log reduction was associated with significantly inferior RFS (3-year RFS rates 24.2% [95% CI 11.0%–53.3%] vs. 48.0% [95% CI 24.6%–93.8%], *p* = 0.001) and higher CIR (3-year CIR rates 71.4% vs. 42.0%, *p* = 0.002) compared to post-induction *IDH2*-R140 MRD > 2 log reduction (n = 38), while *IDH1*-R132 mutation (n = 18) or *IDH2*-R172 mutation (n = 8) MRD had no impact on RFS and CIR.

### 3.7. Outcome Impact of IDH1/2 LNA-qPCR MRD during Consolidation

For CR patients after one or two cycles of consolidation chemotherapy, we did not observe significant differences in RFS (3-year RFS 56.1% [95% CI 40.1%–78.5%] vs. 31.1% [95% CI 16.8%–57.9%], *p* = 0.07, Figure 6d) and OS (3-year OS 59.5% [95% CI 44.0%–80.4%] vs. 54.8% [95% CI 37.3%–80.5%], *p* = 0.9, Figure 6e) between patients with *IDH1/2* LNA-qPCR MRD > 2 log reduction and ≤ 2 log reduction. The CIR was significantly higher for patients with *IDH1/2* LNA-qPCR MRD ≤ 2 log reduction than patients with *IDH1/2* LNA- qPCR MRD > 2 log reduction (3-year CIR 68.8% vs. 42.3%, *p* = 0.019; Figure 6f)

Based on *IDH1/2* mutation subtypes, *IDH2*-R140 MRD ≤ 2 log reduction during consolidation was associated with a trend of inferior RFS (3-year RFS rates 26.8% [95% CI 10.9%–66.0%] vs. 44.2% [95% CI 24.7%–79.1%], *p* = 0.06) and a significantly higher CIR rate (3-year CIR rates 73.2% vs. 44.8%, *p* = 0.013) compared to *IDH2*-R140 MRD > 2 log reduction during consolidation (n = 37), while *IDH1*-R132 mutation (n = 17) or *IDH2*-R172 mutation (n = 8) had no impact on RFS and CIR.

## 4. Discussion

Our cohort of 88 adult patients with *IDH1/2-*mutated AML showed that the concordance rate of *IDH1/2* LNA-qPCR MRD with remission status was 79.5%. There was a positive correlation (r_s_ = 0.68) and a substantial agreement (81%, *K* = 0.74) between *IDH1/2* LNA-qPCR and *NPM1* RT-qPCR MRD. The median difference in MRD results was 2 logs between *IDH1/2* LNA-qPCR and *NPM1* RT-qPCR. Patients with *FLT3* mutation or younger age have a higher concordance rate between *IDH1/2* LNA-qPCR MRD, remission status, or *NPM1* RT-qPCR MRD. At the end of induction and during consolidation, *IDH1/2* LNA-qPCR MRD had a significant impact on CIR or RFS.

Because of the single nucleotide substitution of *IDH1/2* mutations, the sensitivity and specificity of *IDH1/2*-directed MRD are limited if there is a small proportion of *IDH1/2* mutations in the wild-type background. *IDH1/2-*targeted MRD using ddPCR assays reached a sensitivity of 0.1% due to the problem of a relatively high background observed in negative controls, which consisted of false-positive droplets [19]. LNA primers could block the wild-type allele, avoid the false-positive signals resulting from polymerase errors during PCR amplification steps, and allow for higher sensitivity and specificity of *IDH1/2* LNA-qPCR [24,31]. Previous studies have applied diverse methods to detect *IDH1/2-*targeted residual disease by using NGS, ddPCR, or LNA-qPCR. The sensitivity of *IDH1/2* MRD was reported to be 0.1%–0.2% by ddPCR [18,19,32], < 0.001%–1% by NGS [20,21,22], and 10^−4^ by LNA-qPCR [24]. LNA-qPCR assay for *IDH1/2* mutations theoretically has a sensitivity of 1 × 10^−4^, allowing for a deeper evaluation of molecular response. However, we observed a low copy number background of *IDH1/2* mutations by LNA-qPCR in normal BM samples, especially for *IDH1*-R132C (c.394C > T) and *IDH1*-R132H (c.395G > A). There was also a higher mean variant allele fraction of *IDH1*-R132C and *IDH1*-R132H than other *IDH1/2* mutant types detected by ddPCR assay on gDNA extracted from *IDH1/2* wild-type pooled blood lymphocytes in the study by Ferret et al. [19]. The low copy number background of *IDH1/2* LNA-qPCR in normal BM samples seems to be related to the mutant subtype rather than age (Figure S1). Clonal hematopoiesis is unlikely to be the cause of the low copy number background of *IDH1/2* mutants. A possible explanation for this background noise is PCR-induced substitution error caused by cytosine deamination and increased C > T and G > A transition errors, which exhibit strong sequence context dependency [33,34].

We observed a persistently high level of *IDH1/2* mutation at CR in 20% of *IDH1/2*-mutated patients. The discrepancy of persistent *IDH1/2* LNA-qPCR MRD in CR might be attributed to clonal hematopoiesis in pre-leukemic hematopoietic stem cells, which provide a potential reservoir for leukemic progression [35,36]. This hypothesis is supported by the observation of persistent *IDH1*-R132H and *DNMT3A* mutation, loss of *STAG2* mutation at CR, and acquisition of *CEBPA* mutation in one patient in this study. Previous studies reported that persistent *IDH1/2* mutants in AML patients at CR occurred in 39–62% by NGS and 7% by ddPCR [19,20,21]. We found that *NPM1* RT-qPCR MRD showed a median difference of two log reductions compared with *IDH1/2* LNA-qPCR MRD in AML. Low copy number false-positive *IDH1/2* LNA-qPCR background due to PCR-induced substitution errors of *IDH1/2* mutant and detection of gDNA by *IDH1/2* LNA-qPCR rather than cDNA by *NPM1* RT-qPCR, where a single cell may contain multiple copies of RNAs, and low NCN of *IDH1/2* mutants at initial diagnosis might all contribute to the limited sensitivity of *IDH1/2* LNA-qPCR. Due to this, RT-qPCR MRD targeting *NPM1* mutant rather than *IDH1/2* mutant is recommended for AML patients with both *NPM1* and *IDH1/2* mutations.

The use of *FLT3* mutations as a genetic marker for MRD is controversial. This is due to unstable *FLT3* mutations that are acquired or lost during the disease course of AML [37,38]. It lacks standardization of the detection methods for *FLT3* MRD regarding mutation types covered and limited sensitivity [39,40,41]. The ELN MRD working party recommends that the use of *FLT3* mutations is appropriate in combination with other additional MRD markers [26]. NGS-MRD for multiple genetic markers is not widely available. Only one of 25 (4.0%) AML patients with concurrent *IDH1/2* and *FLT3* mutations had a discrepancy between *IDH1/2* mutant MRD and remission-relapse status. Based on this observation, *IDH1/2* mutation might be a helpful surrogate or additional genetic marker for MRD monitoring in patients with concurrent *FLT3* mutation and wild-type *NPM1*.

The loss of *IDH1/2* mutation at relapse of AML has been rarely reported. Brambat et al. reported the loss of *IDH2* mutation at relapse in one of 14 post-transplantation AML patients [18]. All *IDH1/2-*mutated AML patients who had achieved CR retained *IDH1/2* mutant at relapse. The small number of cases detected for *IDH1/2*-MRD at relapse in this cohort might preclude the finding of *IDH1/2* disappearing at relapse. Whether *IDH1/2* mutation could be a stable genetic MRD marker will need to be validated in a larger cohort. The stability of *NPM1* mutation, a consensus recommended MRD marker for AML, was reported to be 99%–100% in large cohorts [14,42], but the loss of *NPM1* mutation at relapse has been observed in 7% to 25% of AML patients in smaller series [17,43,44,45,46,47]. Because of the marked genetic heterogeneity of AML, no single genetic MRD marker can be applied to all patients. As the role of MRD in the management of AML patients is becoming more and more important, *IDH1/2* mutation might be a surrogate MRD marker for a subset of AML patients without recurrent fusion genes or *NPM1* mutations.

A large cohort study of the French ALFA group reported that *IDH1/2* mutant allele burden < 0.2% by ddPCR after induction was a significant predictor of prolonged event-free survival [19]. The MD Anderson Cancer Center study showed that persistent *IDH1/2* mutation in remission detected by NGS at a sensitivity of 1% predicted relapse in multivariate analyses [20]. The results of this study further support the impact of *IDH1/2* MRD on relapse. In a recent study reported by Bill et al., *IDH1*-R132 and *IDH2*-R172 MRD positivity in remission at HSCT was associated with an increased risk of relapse, while *IDH2*-R140 mutations did not [48]. We observed that *IDH1/2* LNA-qPCR MRD elevations preceded frank hematological relapse and confirmed that *IDH1/2* LNA-qPCR MRD after induction therapy and during consolidation could predict relapse in AML patients but did not significantly predict OS. However, we found that *IDH2*-R140 MRD positivity post-induction and during consolidation could predict an increased risk of relapse, whereas *IDH1*-R132 or *IDH2*-R172 did not.

Studies showed that early pre-emptive interventions might improve the outcomes of AML patients in CR with persistent positive fusion genes or *NPM1* mutation [49,50]. However, the feasibility, outcome prediction, and guidance of pre-emptive therapy based on *IDH1/2*-MRD in AML patients was rarely investigated and thus cannot be recommended, mainly due to the concern of *IDH1/2* mutants as pre-leukemic clones as well as the possibility of loss or gain of *IDH1/2* mutants at relapse [26]. For patients with *IDH1/2* MRD > 2 log reduction at the end of induction, the *IDH1/2* MRD correlated with *NPM1* MRD and morphological remission status, and the loss of *IDH1/2* mutation at relapse rarely occurred in the current and previous studies [18,19]. Patients with a deep *IDH1/2* molecular response after achieving CR will benefit from serial *IDH1/2* MRD monitoring to identify early relapse. On the other hand, patients with persistently high *IDH1/2* MRD at first CR should not be followed by *IDH1/2-*targeted MRD alone. It is still unclear whether early pre-emptive therapy will benefit AML patients in CR who have increasing *IDH1/2* mutant MRD during follow-up, which requires further evaluation in prospective clinical trials.

## 5. Conclusions

Our study showed that the correlation and stability of *IDH1/2* MRD by LNA-qPCR was good in patients who had concordantly reduced *IDH1/2* MRD in morphologic CR. *IDH1/2* LNA-qPCR MRD > 2 log reduction at first CR and during consolidation is associated with improved CIR or RFS. *IDH1/2* LNA-qPCR MRD has the potential to become a clinical tool to monitor deep treatment response and predict relapse for *IDH1/2* mutated AML patients, especially for those who are younger, with reduced *IDH1/2* MRD at first CR, or concurrent *FLT3* mutation.

## Figures and Tables

**Table 1:** Probes, forward primers, and reverse primers.

Target	Reverse Primer (5′-3′)	Forward Primer (5′-3′)	Probe (5′-3′)
*IDH1*-R132C	R-LNA-0: GATCCCCATAAGCATGACA	CGGTCTTCAGAGAAGCCATT	FAM-ATGGGTAAAACCTATCATC-MGB
*IDH1*-R132H	R-LNA-0: CTTGATCCCCATAAGCATGAT		
*IDH1*-R132S	R-LNA-0: GATCCCCATAAGCATGACT		
*IDH1*-R132G	R-LNA-0: GATCCCCATAAGCATGACC		
*IDH1*-R132L	R-LNA-0: CTTGATCCCCATAAGCATGAA		
*IDH2*-R140Q	R-LNA-0: GTCCCCCCCAGGATGTTCT	AGTTCAAGCTGAAGAAGATGTGG	FAM-AGTCCCAATGGAACTA-MGB
*IDH2*-R140W	R-LNA-0: GTCCCCCCCAGGATGTTCCA		
*IDH2*-R172K	GTCGCCATGGGCGTGCT	TCCCACGCCTAGTCCCTGGCTG	FAM-AGCCCATCACCAT-MGB
*ALB*	CTCTCCTTCTCAGAAAGTGTGCATAT	TGAAACATACGTTCCCAAAGAGTTT	FAM-TGCTGAAACATTCACCTTCCATGCAGA -TAMRA

R-LNA-0, a locked nucleic acid was fixed in the last 3′ nucleotide of the reverse primers.

**Table 2:** Clinical and genetic characteristics of *IDH1/2-*mutated AML patients.

Variables	All (N = 88)	*IDH1* (N = 25)	*IDH2* (N = 63)	*p*
Median age, range (years) *	54 (18–86)	54 (36–68)	55 (18–86)	0.582
**Gender**				0.587
Female, n (%)†	47 (53)	15 (60)	32 (51)	
Male, n (%)†	41 (47)	10 (40)	31 (49)	
Hemoglobin, range (g/dL) *	7.9 (3.7–14.3)	8.1 (3.7–12.6)	7.8 (3.8–14.3)	0.627
White blood cell count, range (× 10^9^/L) *	12.8 (0.7–354.6)	11.2 (0.9–162.0)	13.2 (0.7–355.0)	0.701
Platelet count, range (× 10^9^/L) *	66 (0–382)	47 (7–194)	70 (0–382)	0.134
Circulating blasts, range (%) *	47 (0–99)	80 (6–99)	37 (0–99)	0.005
Marrow blasts, range (%) *	67 (19–96)	86 (20–96)	61 (19–94)	0.627
**2022 WHO AML classification †**				
AML with *NPM1* mutation	42 (48)	11 (44)	31 (49)	
AML with *CEBPA* mutation	2 (2)	0 (0)	2 (3)	
AML with TP53 mutation	1 (1)	1 (4)	0 (0)	
AML, myelodysplasia-related	18 (20)	5 (20)	13 (21)	
AML with minimal differentiation	1 (1)	0 (0)	1 (2)	
AML without maturation	6 (7)	4 (16)	2 (3)	
AML with maturation	16 (18)	3 (12)	13 (21)	
Acute myelomonocytic leukemia	2 (2)	1 (4)	1 (2)	
**Cytogenetic risk †**				0.755
Normal, n (%)	56 (64)	14 (56)	42 (67)	
Intermediate risk, n (%)	15 (17))	4 (16)	11 (17))	
Adverse risk, n (%)	8 (9)	3 (12)	5 (8)	
Unknown, n (%)	9 (10)	4 (16)	5 (8)	
**2022 ELN risk group †**				0.384
Favorable, n (%)	31 (35)	6 (24)	25 (40)	
Intermediate, n (%)	32 (36)	9 (36)	23 (37)	
Unfavorable, n (%)	22 (25)	8 (32)	14 (22)	
Unknown	3 (4)	2 (8)	1 (2)	
**Treatment †**				0.534
Standard chemotherapy, n (%)	84 (95)	24 (96)	60 (95)	
Azacitidine, n (%)	2 (2)	1 (4)	1 (2)	
Low-dose cytarabine, n (%)	2 (2)	0 (0)	2 (3)	
***IDH1/2* mutation †**				
*IDH1-*R132C/H/G/S/L, n (%)	25 (28)	11/9/2/2/1	-	
*IDH2-*R140Q/W, n (%)	51 (58)	-	49/2 (81)	
*IDH1-*R172K, n (%)	12 (14)	-	12 (19)	
Median *IDH1/2* NCN at diagnosis, range (/*ALB* × 10^6^)	53,228 (87–980,686)	80,920 (31,343–980,686)	46,156 (87–247,796)	<< 0.001
**Concurrent gene mutations †**				
*FLT3*-ITD, n (%)	20 (23)	8 (32)	12 (19)	
*FLT3*-TKD, n (%)	6 (7)	2 (8) #	4 (6)	
*EZH2,* n (%)	1 (1)	0 (0)	1 (2)	
*RUNX1*, n (%)	7 (8)	4 (16)	3 (5)	
*ASXL1*, n (%)	4 (5)	1 (4)	3 (5)	
*STAG2*, n (%)	4 (5)	1 (4)	3 (5)	
*SF3B1*, n (%)	1 (1)	0 (0)	1 (2)	
*BCOR*, n (%)	3 (4)	1 (4)	2 (3)	
*SRSF2*, n (%)	1 (1)	0 (0)	1 (2)	

* Data are expressed as median (minimum-maximum). † Data are expressed as number of patients (percentage). # one patient with both *FLT3*-ITD and *FLT3*-TKD. NCN, normalized copy number; ELN, European LeukemiaNet; ITD, internal tandem duplication; TKD, tyrosine kinase domain.

**Table 3:** Clinical characteristics and the correlation between *IDH1/2* qPCR MRD and hematologic remission status.

Factors	With Correlation (n = 69)	Without Correlation (n = 19)	*p*
Age, range (years) *	53 (18–86)	61 (23–74)	0.025
NCN at diagnosis (/*ALB* × 10^6^) *	53,281 (1252–980,686)	44,929 (87–181,539)	0.903
Marrow blasts, range (%) *	71 (19–96)	60 (23–94)	0.231
WBC, range (× 10^9^/L) *	20.4 (0.7–355.0)	10.3 (1.4–162.0)	0.271
Hemoglobin, range (g/dL) *	7.8 (3.7–13.6)	8.1 (4.3–14.3)	0.520
Platelet, range (× 10^9^/L) *	65 (7–382)	69 (0–201)	0.594
Circulating blasts, range (%) *	52 (0–98)	44 (0–99)	0.294
*FLT3*-ITD/TKD ^†^			0.012
Positive	24	1	
Negative	45	18	
*NPM1* mutation ^†^			0.458
Positive	31	11	
Negative	38	8	

* Data are expressed as median (minimum-maximum). † Data are expressed as number of patients. NCN, normalized copy number; qPCR, quantitative RT-PCR; MRD, measurable residual disease; CR, complete remission; ITD, internal tandem duplication; TKD, tyrosine kinase domain.

## Data Availability

The datasets used and/or analyzed in the current study are available from the corresponding author on reasonable request.

## Supplementary Materials

The following supporting information can be downloaded at: https://www.mdpi.com/article/10.3390/cancers14246205/s1, Table S1. *IDH1/2* LNA-qPCR of 19 normal bone marrow samples from healthy donors. Figure S1. *IDH1/2* LNA-qPCR of 19 normal bone marrow samples from healthy donors according to age. Figure S2. MRD kinetics of individual patients with good correlation between *IDH1* LNA-qPCR MRD, *NPM1* RT-qPCR, or remission-relapse status. Figure S3. MRD kinetics of individual patients with discordant *IDH1* LNA-qPCR MRD, *NPM1* RT-qPCR MRD, or remission status. Figure S4. MRD kinetics of individual patients with good correlation between *IDH1* LNA-qPCR MRD and hematological remission-relapse status. Figure S5. MRD kinetics of individual patients with persistent high *IDH1* LNA-qPCR and relapse refractory status. Figure S6. MRD kinetics of individual patients with good correlation between *IDH2* LNA-qPCR MRD, *NPM1* RT-qPCR, or remission-relapse status. Figure S7. MRD kinetics of individual patients with discordant *IDH2* LNA-qPCR MRD, *NPM1* RT-qPCR MRD, and hematological remission status. Figure S8. MRD kinetics of individual patients with good correlation between *IDH2* LNA-qPCR MRD and hematological remission-relapse status. Figure S9. MRD kinetics of individual patients with persistent high *IDH2* LNA-qPCR and relapse refractory status. Figure S10. Summary of MRD kinetics according to the concordance between *IDH1/2* LNA-qPCR MRD and the disease status.

## Abbreviations

BM: Bone marrow; IDH: Isocitrate dehydrogenase; MRD: Measurable residual disease; ddPCR: Digital PCR; NGS: Next-generation sequencing; LNA: Locked nucleic acid; qPCR: Quantitative Real-Time PCR; CR: Complete remission; ELN: European LeukemiaNet; gDNA: Genomic DNA; *ALB*: Albumin; NCN: Normalized copy number; RT-qPCR: Quantitative Real-Time reverse transcriptase PCR; OS: Overall survival; RFS: Relapse-free survival; CIR: Cumulative incidence of relapse; HSCT: Allogeneic hematopoietic stem cell transplantation; ITD: Internal tandem duplication; TKD: Tyrosine kinase domain.
