# Supplementary material for: Measurable Residual Disease Monitoring by Locked Nucleic Acid Quantitative Real-Time PCR Assay for IDH1/2 Mutation in Adult AML
Source: Cancers (Basel). 2022 Dec 15;14(24):6205. doi: 10.3390/cancers14246205 (PMC9777301; doi:10.3390/cancers14246205)

Table S1 *IDH1/2* LNA-qPCR of 19 normal bone marrow samples from healthy donors

***IDH1/2* mutant type**	**Copy**	**S.D. (Copy)**	**NCN (*IDH/ALB*x10^6)**	**S.D (NCN)**
R132C (c.394C>T)	73.12	28.70	1119.43	569.22
R132G (c.394C>G)	0.01	0	0.20	0.29
R132H (c.395G>A)	33.19	16.14	462.23	181.04
R132L (c.395G>T)	0.90	1.10	11.33	14.24
R132S (c.394C>A)	5.72	2.74	77.22	37.44
R140Q (c.419G>A)	14.11	25.76	337.32	589.66
R140W(c.418C>T)	4.78	1.88	122.52	39.13
R172K (c.515G>A)	3.73	2.12	81.09	46.63

S.D. standard deviation; NCN: normalized copy number

Figure S1 *IDH1/2* LNA-qPCR of 19 normal bone marrow samples from healthy donors according to age

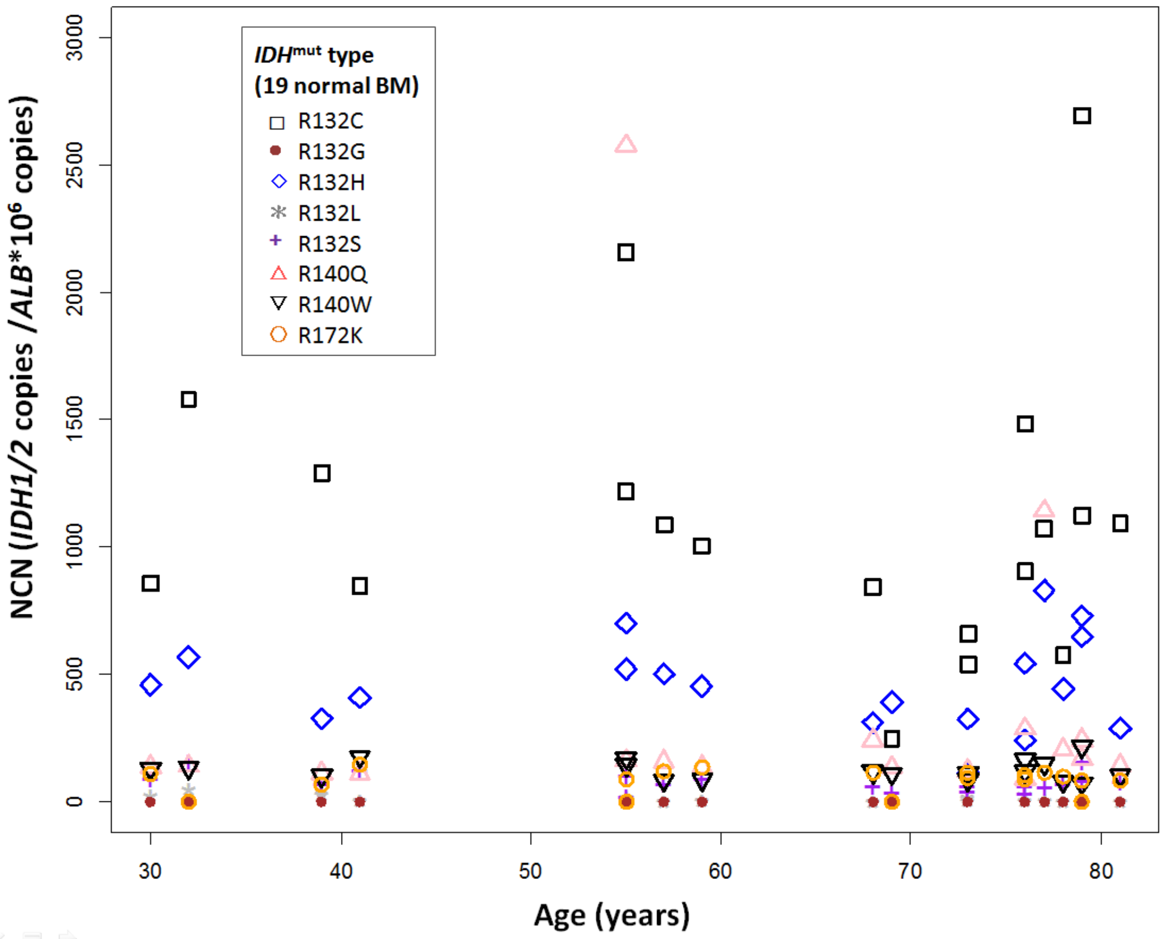

Figure S2 MRD kinetics of individual patients with good correlation between *IDH1* LNA-qPCR MRD, *NPM1* RT-qPCR, or remission-relapse status

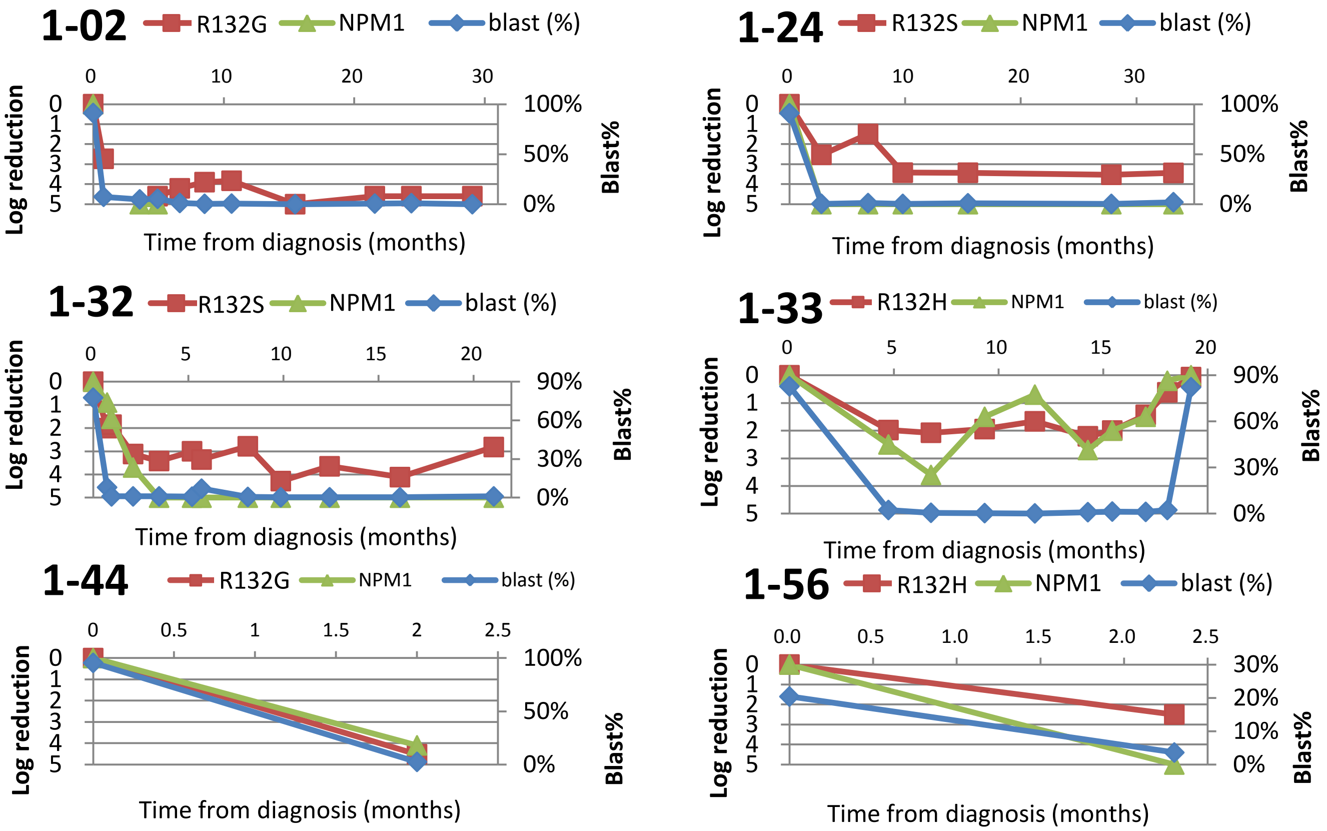

X axis indicating time from diagnosis; left Y axis indicating log reduction of mutated-target gene MRD level compared with that of diagnostic sample; right Y axis Indicating percentage of blasts in bone marrow smear.

Figure S3 MRD kinetics of individual patients with discordant *IDH1* LNA-qPCR MRD, *NPM1* RT-qPCR, or remission status. **(a)** with *NPM1*-MRD; **(b)** without *NPM1*-MRD.

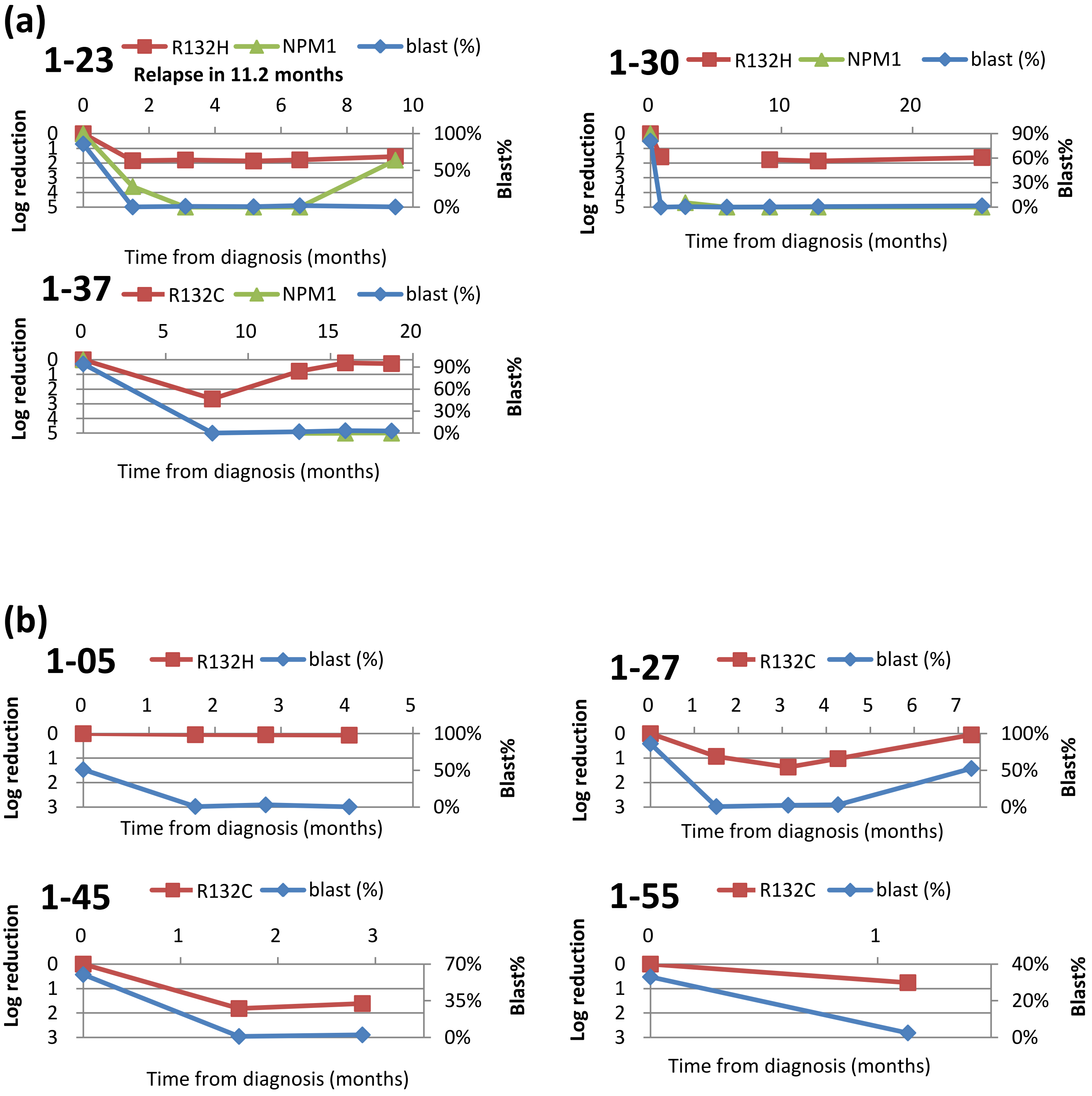

X axis indicating time from diagnosis; left Y axis indicating log reduction of mutated-target gene MRD level compared with that of diagnostic sample; right Y axis Indicating percentage of blasts in bone marrow smear.

Figure S4 MRD kinetics of individual patients with good correlation between *IDH1* LNA-qPCR MRD and hematological remission-relapse status

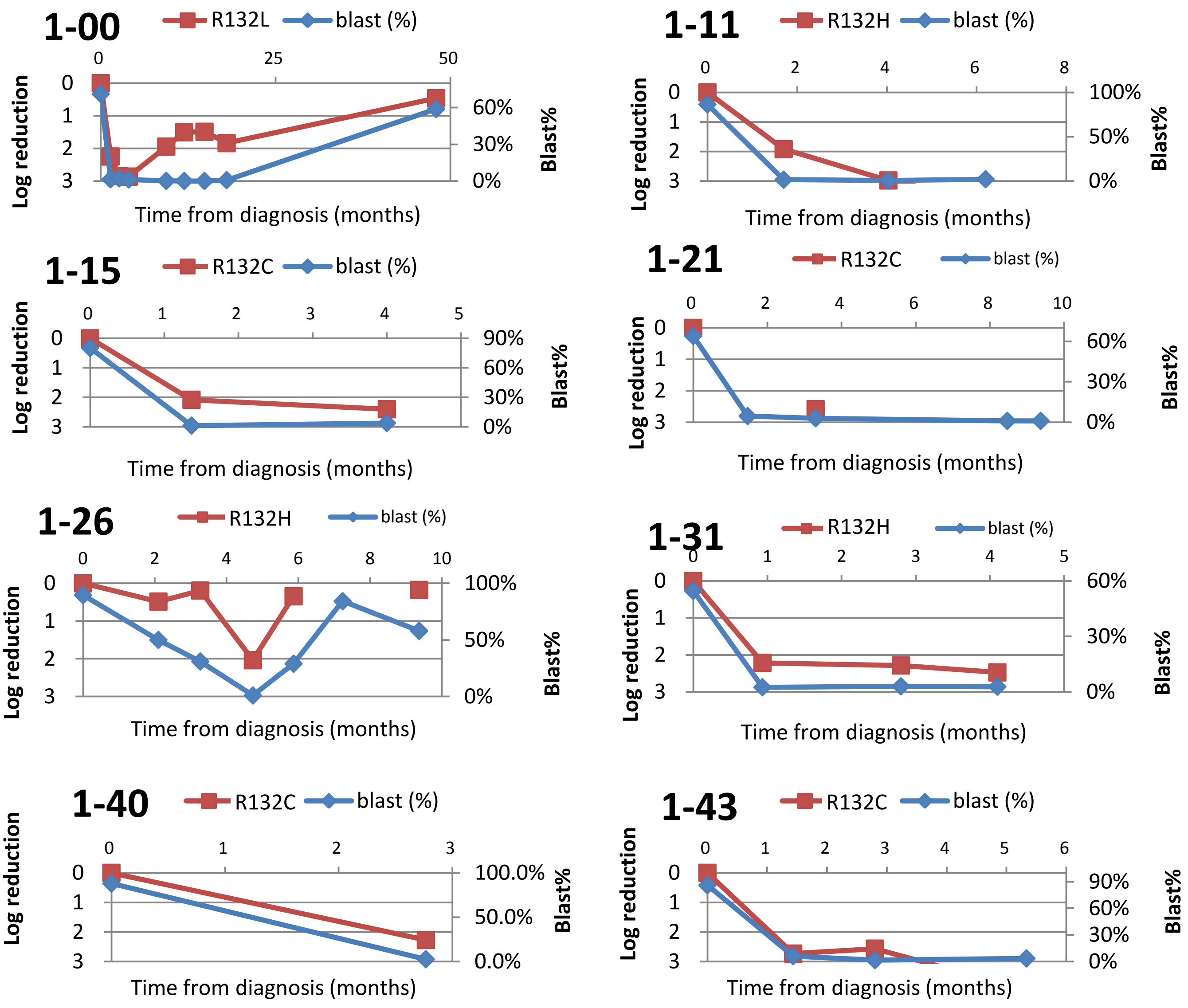

X axis indicating time from diagnosis; left Y axis indicating log reduction of mutated-target gene MRD level compared with that of diagnostic sample; right Y axis Indicating percentage of blasts in bone marrow smear.

Figure S5 MRD kinetics of individual patients with persistent high *IDH1* LNA-qPCR MRD and relapsed or refractory status

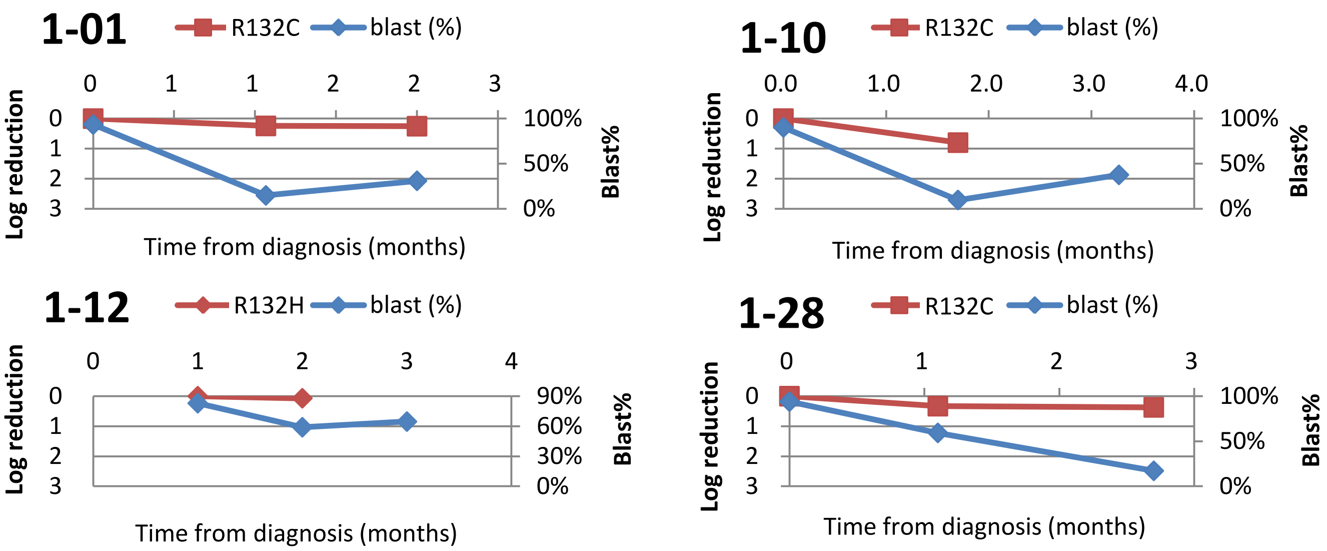

X axis indicating time from diagnosis; left Y axis indicating log reduction of mutated-target gene MRD level compared with that of diagnostic sample; right Y axis Indicating percentage of blasts in bone marrow smear.

Figure S6 MRD kinetics of individual patients with good correlation between *IDH2* LNA-qPCR MRD, *NPM1* RT-qPCR, or remission-relapse status

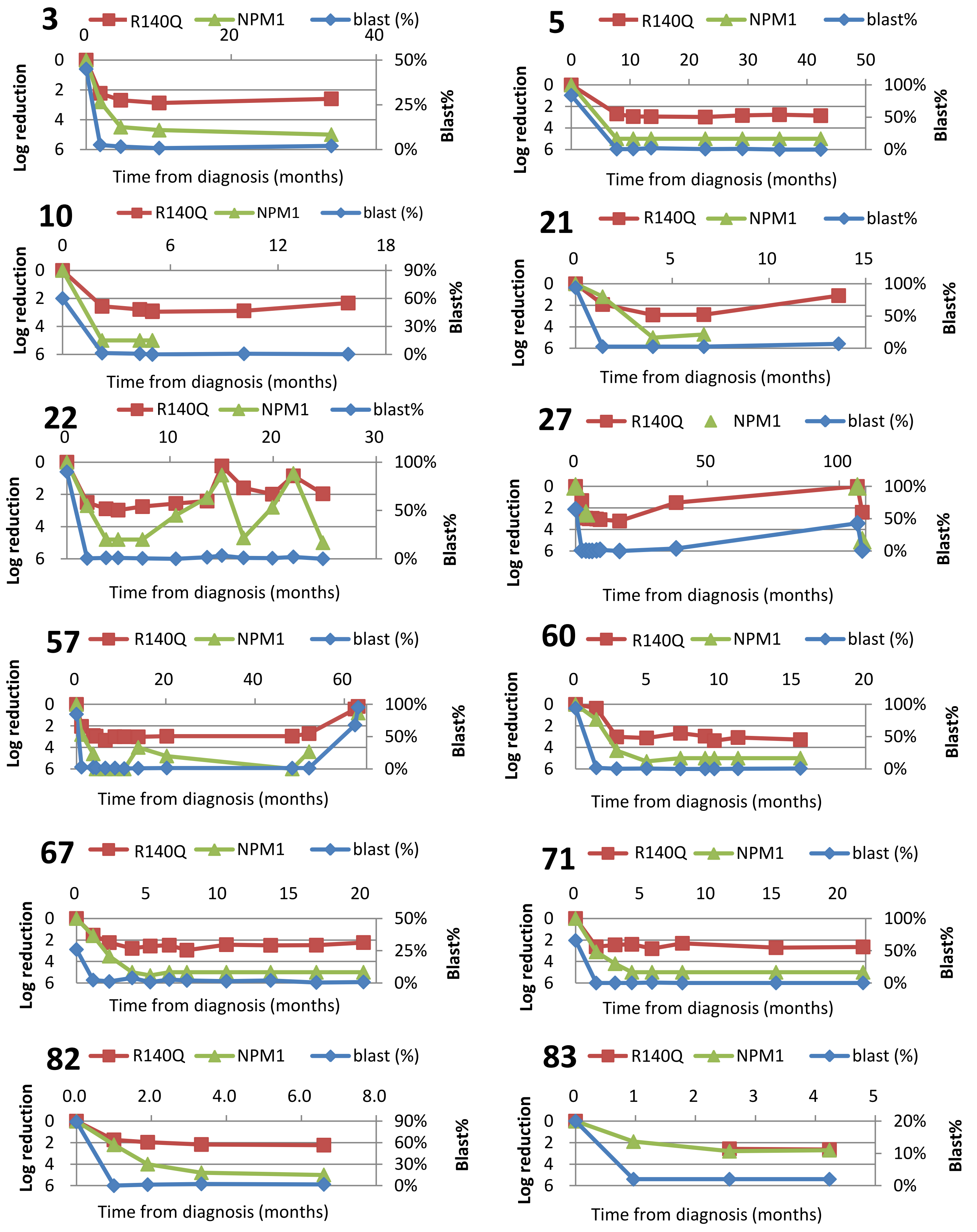

Figure S6 (cont.) MRD kinetics of individual patients with good correlation between *IDH2* LNA-qPCR MRD, *NPM1* RT-qPCR, or remission-relapse status

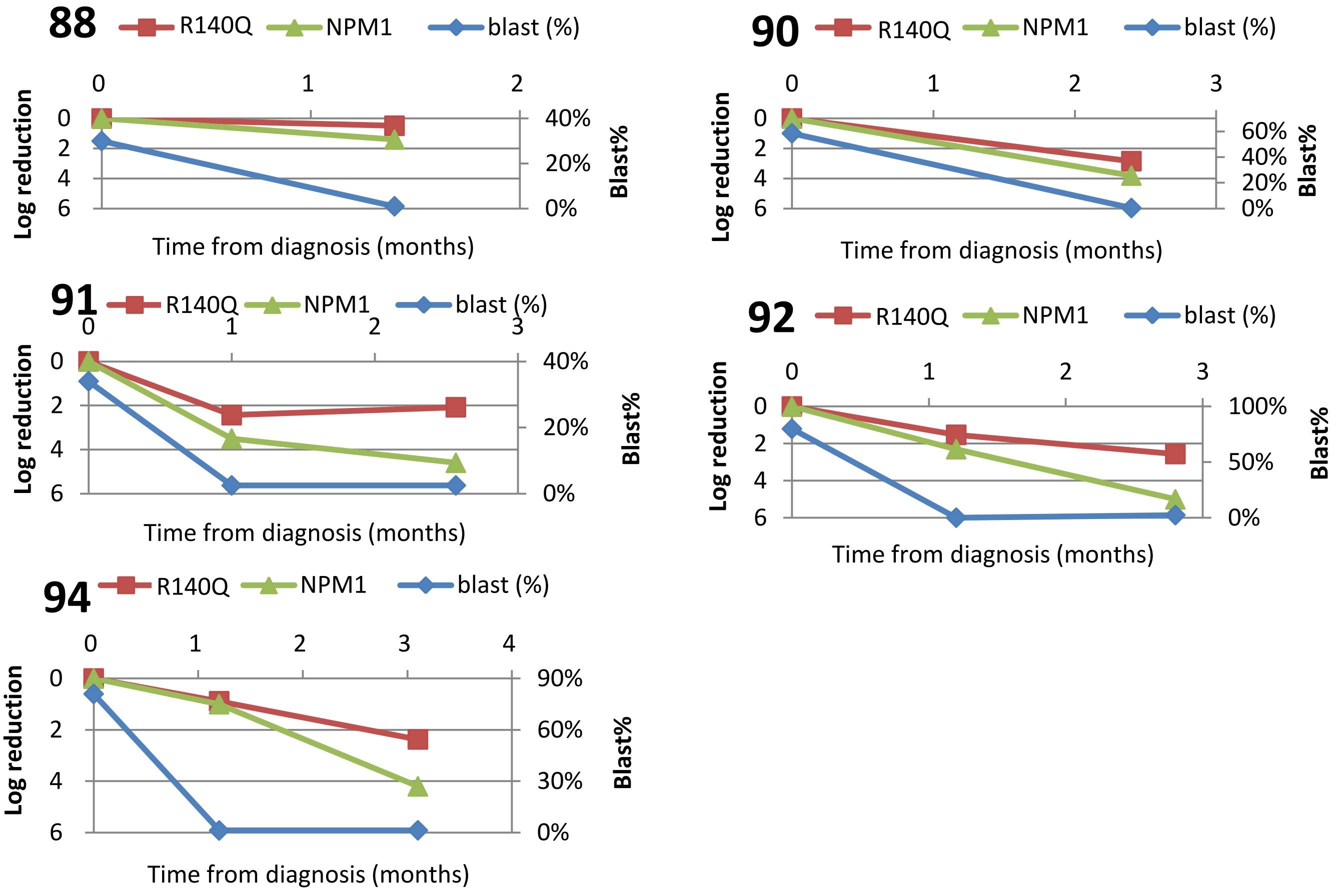

X axis indicating time from diagnosis; left Y axis indicating log reduction of mutated-target gene MRD level compared with that of diagnostic sample; right Y axis Indicating percentage of blasts in bone marrow smear.

Figure S7 MRD kinetics of individual patients with discordant *IDH2* LNA-qPCR MRD, *NPM*1 RT-qPCR MRD, and hematology remission status. **(a)** with *NPM1*-MRD; **(b)** without *NPM1*-MRD.

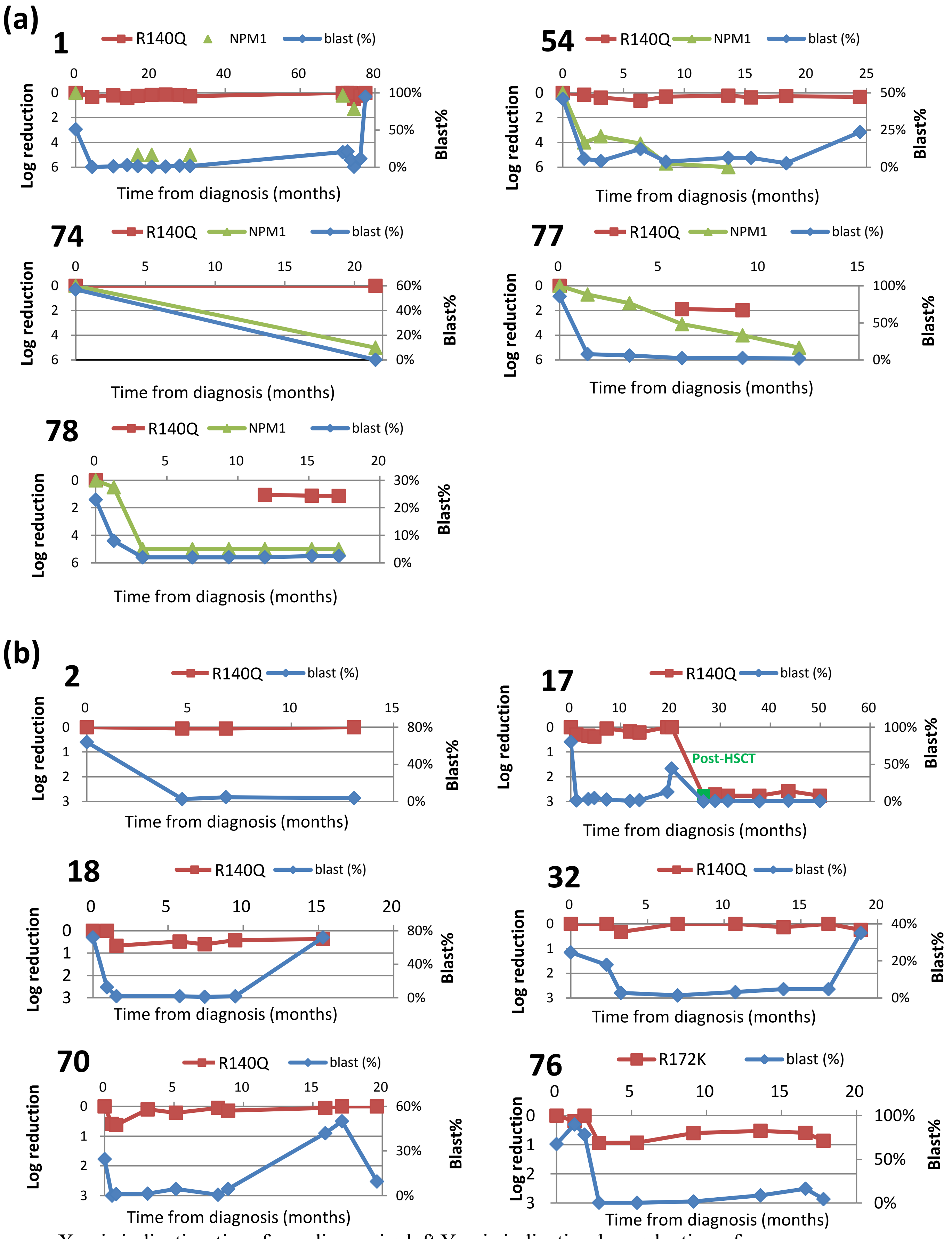

X axis indicating time from diagnosis; left Y axis indicating log reduction of mutated-target gene MRD level compared with that of diagnostic sample; right Y axis Indicating percentage of blasts in bone marrow smear.

Figure S8 MRD kinetics of individual patients with good correlation between *IDH2* LNA-qPCR MRD and hematological remission-relapse status

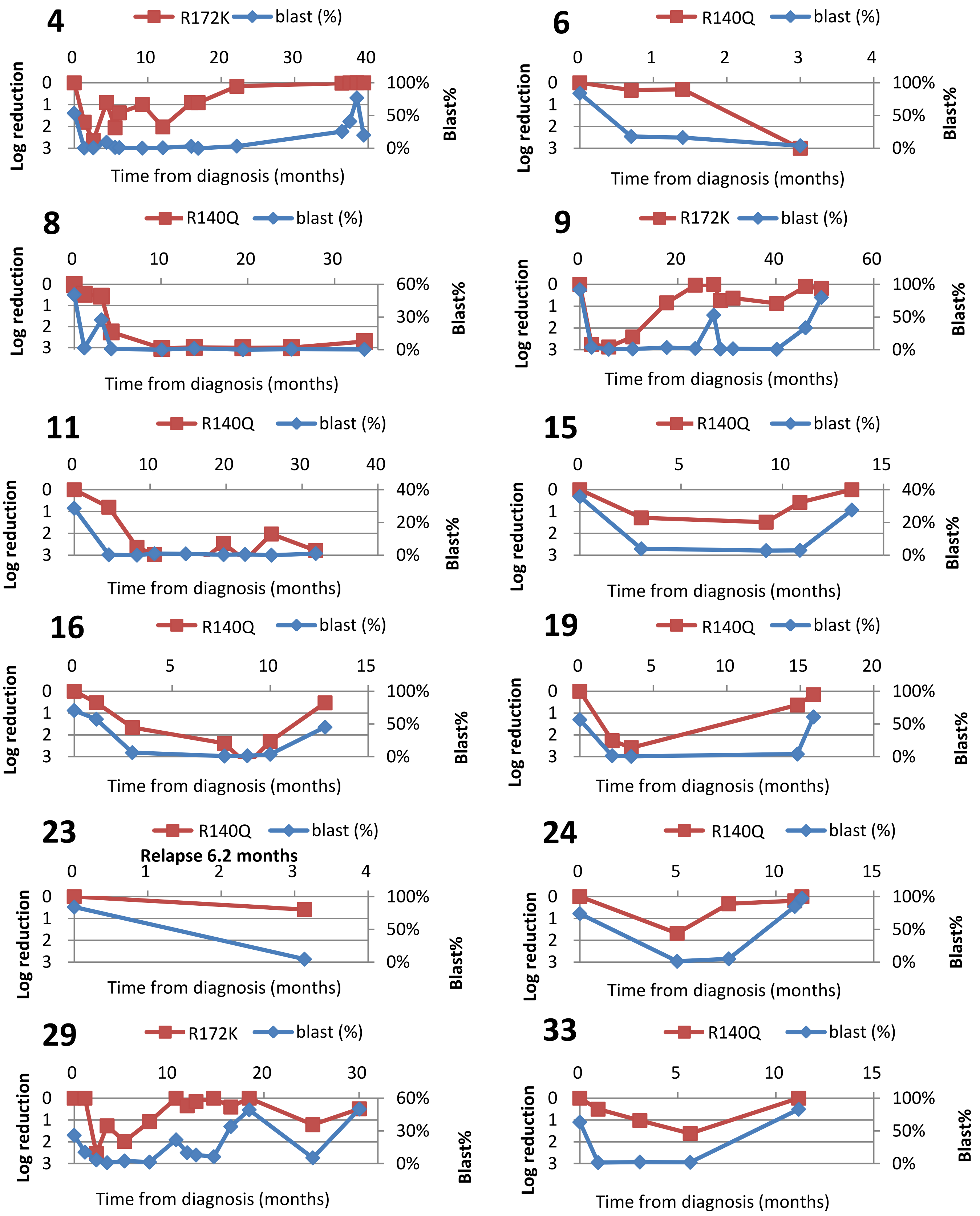

Figure 8 (cont.) MRD kinetics of individual patients with good correlation between *IDH2* LNA-qPCR MRD and hematological remission-relapse status

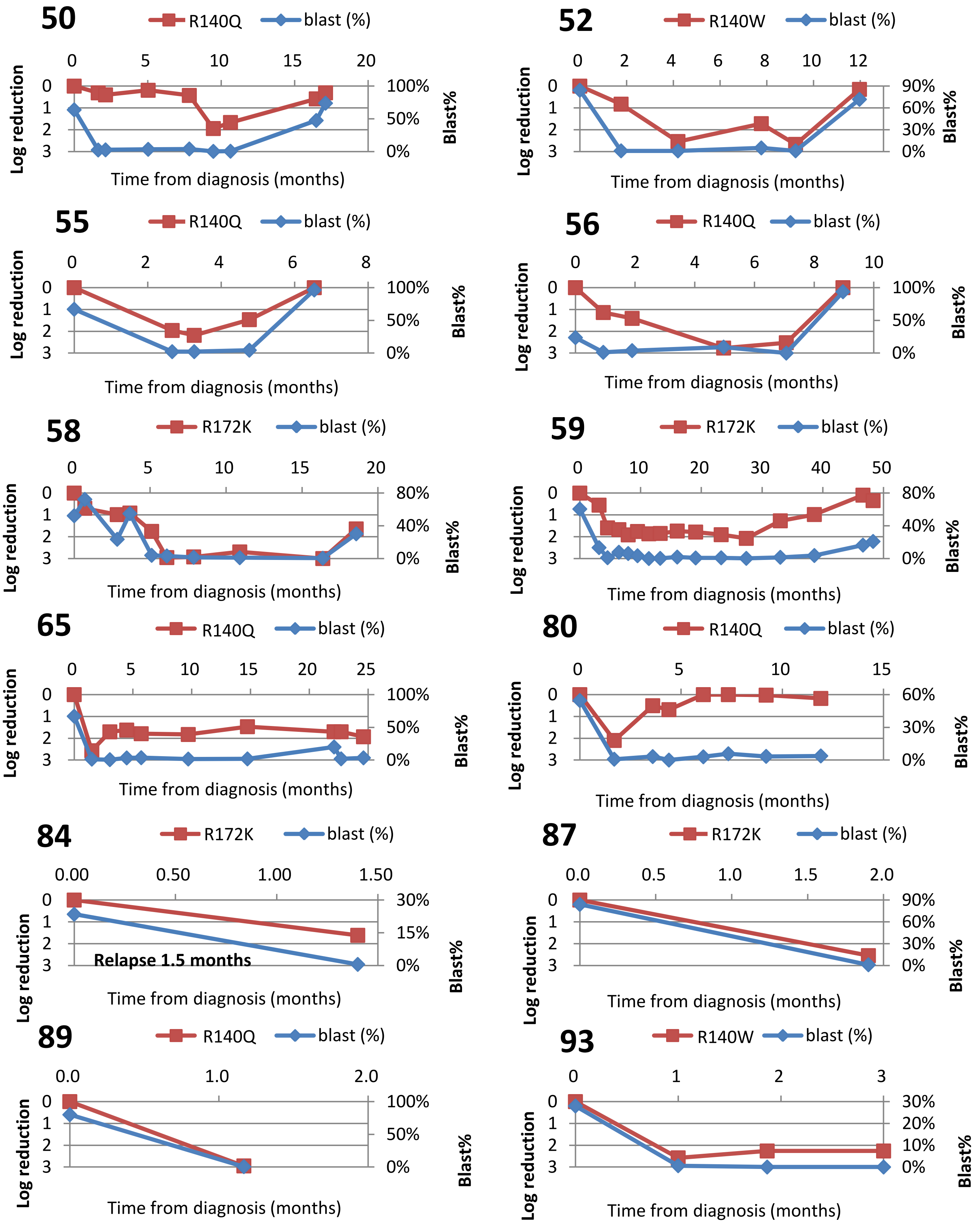

Figure S8 (cont.) MRD kinetics of individual patients with good correlation between *IDH2* LNA-qPCR MRD and hematological remission-relapse status

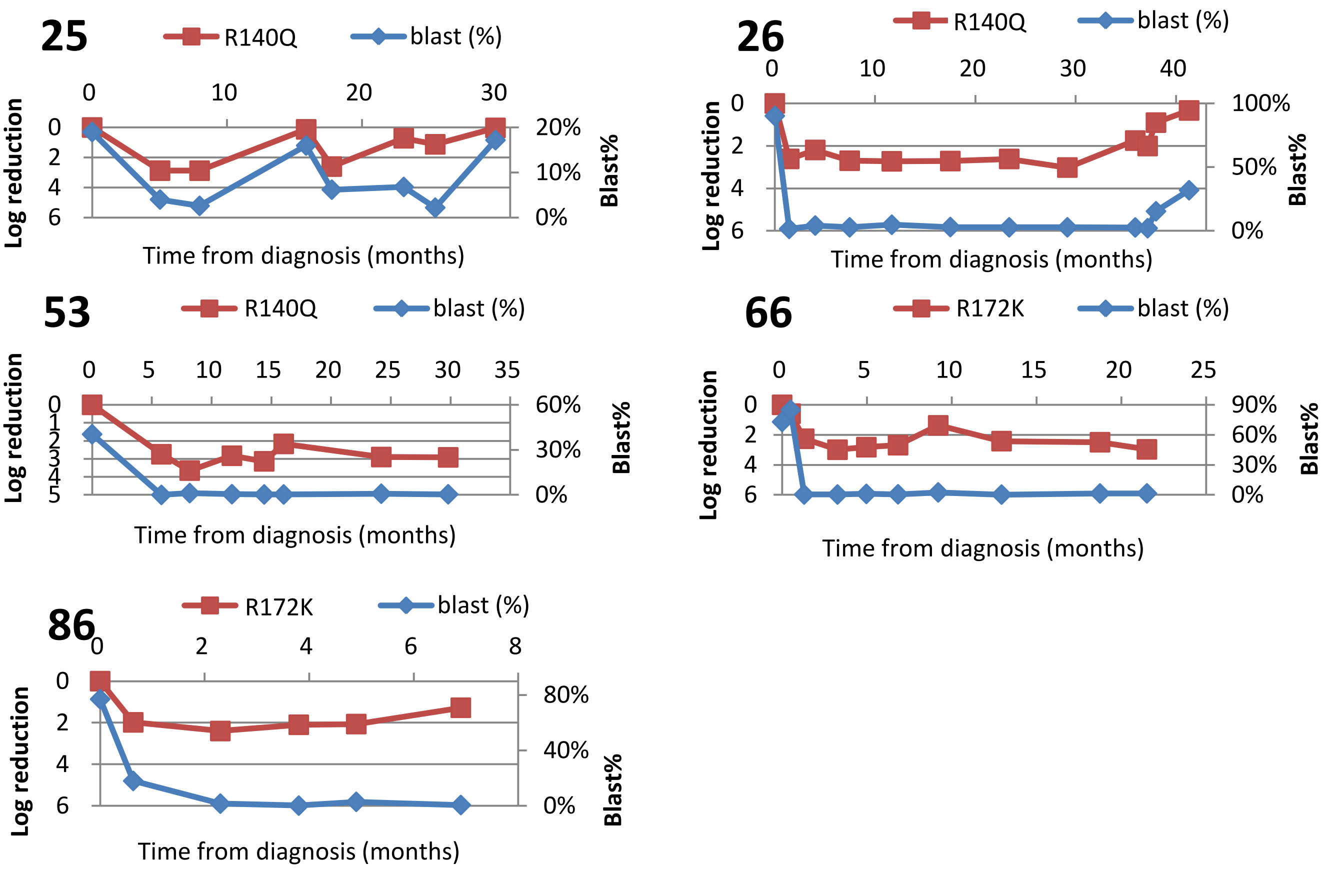

X axis indicating time from diagnosis; left Y axis indicating log reduction of mutated-target gene MRD level compared with that of diagnostic sample; right Y axis Indicating percentage of blasts in bone marrow smear.

Figure S9 MRD kinetics of individual patients with persistent high *IDH2* LNA-qPCR MRD and relapsed refractory status

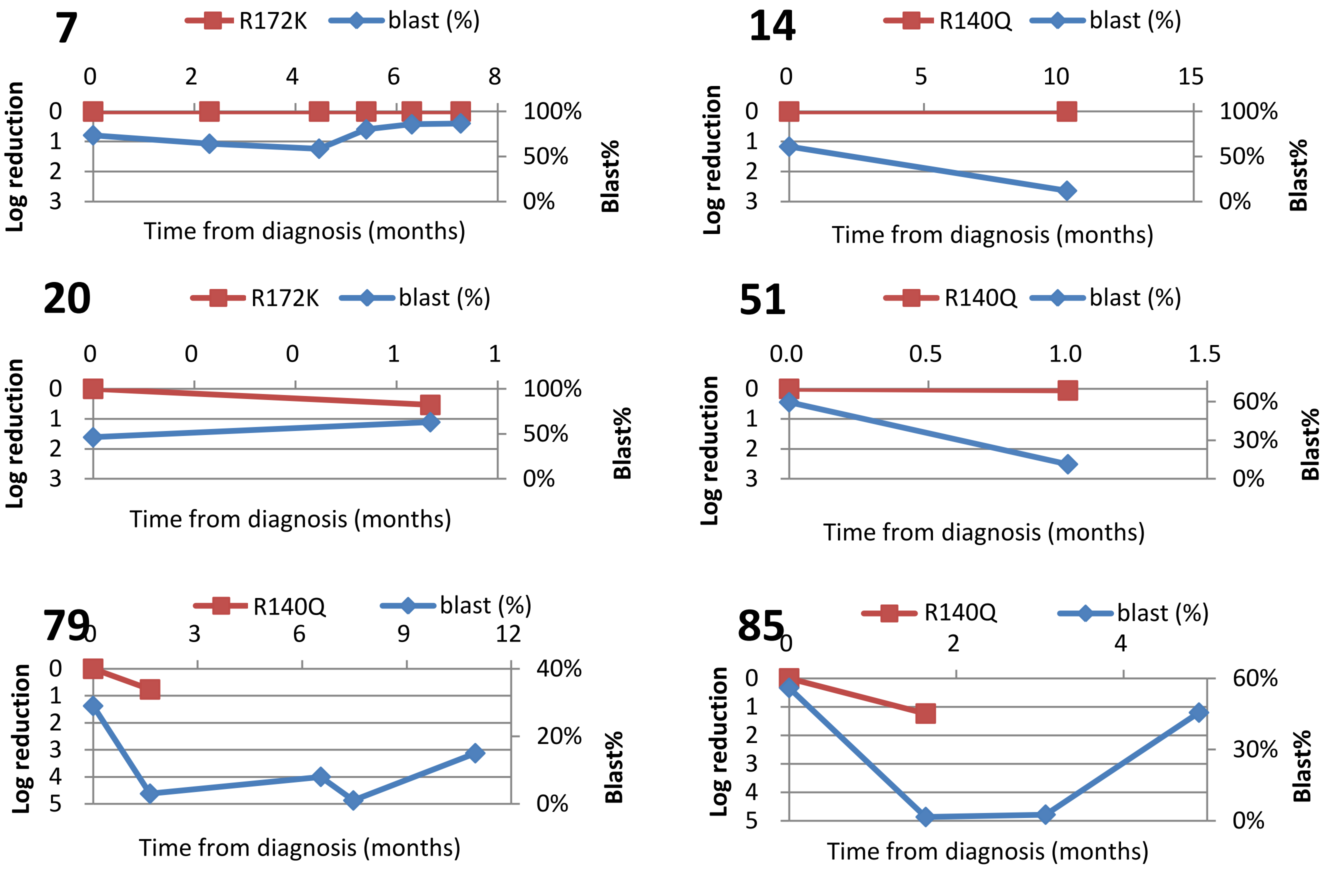

X axis indicating time from diagnosis; left Y axis indicating log reduction of mutated-target gene MRD level compared with that of diagnostic sample; right Y axis Indicating percentage of blasts in bone marrow smear.

Figure S10 Summary of MRD kinetics according to the concordance between *IDH1/2* LNA-qPCR MRD and the disease status. *IDH1/2*-MRD kinetics of **(a)** concordant patients who achieved complete remission and without relapse, **(b)** concordant patients who achieved complete remission and with relapse, **(c)** primary refractory patients, **(d)** patients with persistent high *IDH1/2*-MRD during complete remission.

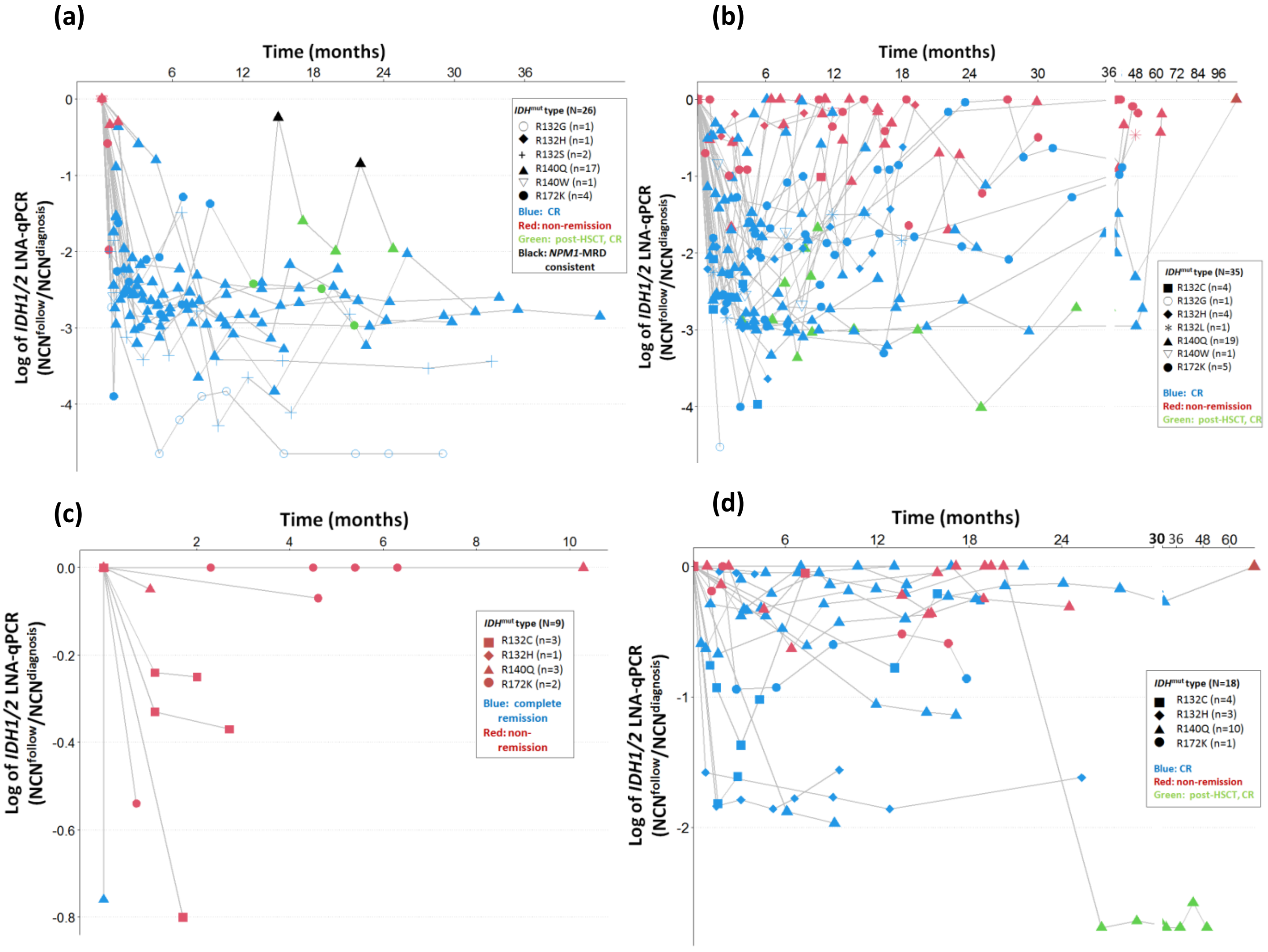

Supplement: Supplementary file 1 [file cancers-14-06205-s001.zip › cancers-2024208-supplementary.pdf]
